# ENHANCING SOCIAL CAPITAL IN OLDER ADULTS: STRATEGIES IN THE ASIAN CONTEXT

**DOI:** 10.1093/geroni/igae098.3839

**Published:** 2024-12-31

**Authors:** On Fung Chan, Terry Lum, Shiyu Lu, Hiu-kwan Chui

**Affiliations:** The University of Hong Kong, Hong Kong, China (People’s Republic); The University of Hong Kong, Hong Kong, China (People’s Republic); The University of Hong Kong, Hong Kong, China (People’s Republic); The University of Hong Kong, Hong Kong, China (People’s Republic)

## Abstract

Research on enhancing social capital among older adults in East Asia often neglects the aspects of trust, norms, and reciprocity, focusing primarily on social participation. Moreover, it frequently overlooks the capacity of older adults for collective action within society. This study addresses these gaps by exploring strategies that enable older adults in Hong Kong to shift from being passive service recipients to active participants in community and collective action. Using a qualitative research design. We conducted focus groups with 43 participants over 60, who were committee members of various socially beneficial groups, including self-initiated and NGO-led groups. These groups engaged in activities such as promoting age-friendliness, ageing in place and ecological conservation. Data were collected through semi-structured interviews and analyzed using deductive and inductive thematic analysis based on Nahapiet and Ghoshal’s framework. The findings identified five key strategies to enhance social capital among older adults: establishing connections with trusted intermediaries, transforming individuals to share collective goals, providing stable platforms for trust-building, facilitating informal interactions to enhance cohesion, and leveraging influential figures to reinforce group norms. These strategies empower older adults and integrate them into the broader social fabric, thereby contributing to collective action. The study provides actionable insights for policymakers and social workers to develop interventions that enhance social capital among older adults, thereby leveraging their potential as active societal contributors.

